# Exploring general practitioners’ perceptions about the primary care gatekeeper role in Indonesia

**DOI:** 10.1186/s12875-020-01365-w

**Published:** 2021-01-04

**Authors:** Joko Mulyanto, Yudhi Wibowo, Dionne S. Kringos

**Affiliations:** 1 Department of Public Health and Community Medicine, Faculty of Medicine, Universitas Jenderal Soedirman, Purwokerto, Indonesia; 2 Department of Public and Occupational Health, Amsterdam UMC, University of Amsterdam; and Amsterdam Public Health Research Institute, Amsterdam, Netherlands

**Keywords:** Primary care, Gatekeeper, Referral behaviour, General practitioner, Perception

## Abstract

**Background:**

In the current healthcare delivery system funded by National Health Insurance (NHI) in Indonesia, the gatekeeper role of primary care services is critical to ensuring equal healthcare access for the population. To be effective, gatekeeping relies on the performance of general practitioners (GPs). However, the perceptions held by Indonesian GPs about their gatekeeper role are not yet well documented. This study describes the self-perceived knowledge, attitudes and performance of Indonesian GPs with respect to the gatekeeper role and explores associated factors.

**Methods:**

We conducted a cross-sectional study of all primary care facilities (*N* = 75) contracted by the regional NHI office in the Banyumas district. The 73 participating GPs completed a written questionnaire that assessed their knowledge, attitudes and performance in relation to the gatekeeper role. Personal and facility characteristics were analysed in a generalised linear model as possible associating factors, as well as for the association between GPs’ knowledge and attitude with performance as gatekeepers.

**Results:**

GPs scored relatively high in the domains of knowledge and performance but scored lower in their attitudes towards the gatekeeper role of primary care. In the full-adjusted model, no factors were significantly associated with the knowledge score. Work experience as GPs, private or civil service employment status and rural or urban location of the primary care facility were linked to attitude scores. Full- or part-time employment and type of facility were factors associated with the performance score. Attitude scores were positively associated with performance score.

**Conclusion:**

GPs in Indonesia are knowledgeable and report that they adequately perform their function as gatekeepers in primary care. However, their attitudes towards the gatekeeper function are less positive. Attitudes and performance with respect to the primary care gatekeeper role are likely influenced more by contextual factors such as location and type of facility than by personal factors. Efforts to address contextual issues could include improvements in practice standards for privately practising physicians and public information campaigns about gatekeeping regulations. Such efforts will be crucial to improving the gatekeeper role of primary care in Indonesia and assuring efficient access to high-quality care for all.

**Supplementary Information:**

The online version contains supplementary material available at 10.1186/s12875-020-01365-w.

## Background

Wide socioeconomic inequalities in secondary healthcare utilisation have been documented in Indonesia [1]. In the wake of the rapid expansion of National Health Insurance (NHI) coverage during recent years, the utilisation of secondary care now depends largely on the functioning of primary care [2]. In the NHI-funded healthcare delivery system, direct access to secondary care is not covered except in emergency cases. NHI beneficiaries must register at a single primary care facility within their district of residence, and that facility acts as a gatekeeper, including the provision of referrals for them to access secondary care [3]. By 2017, around 72% of the Indonesian population, or 177 million people, were covered by NHI, and the health insurance agency had contracted approximately 21,700 primary care facilities to provide beneficiaries with healthcare and gatekeeping services [4]. The numbers continue to grow towards the aim of universal health coverage. The NHI-funded healthcare delivery system in Indonesia is now the largest single-payer system in the world [4].

The role of primary care as gatekeeper is intended to ensure that beneficiaries have access to quality healthcare services while maintaining their efficiency. This is to be achieved via two principal mechanisms. The first is to provide patients with comprehensive health services and continuity of care, which is much easier if patients are registered at a single primary care facility [5]. The second is to deliver healthcare services more efficiently, for instance by curbing the unnecessary use of secondary care [5]. The latter mechanism likely depends on the behaviour of general practitioners (GPs), who share key responsibility for carrying out the gatekeeper function on a daily basis. Physicians’ perceptions of their role and responsibilities in the healthcare system – including their knowledge, attitudes and practices – have been shown to influence whether they can optimally fulfil their role as physicians [6]. Arguably, differences in how GPs perceive their gatekeeping role may affect the performance of primary care services as gatekeepers. Factors such as variations in referral behaviour could create disparities in the utilisation of secondary healthcare.

Studies on how primary care performs its role of gatekeeper are still limited in Indonesia, and particularly from the GP perspective. A study from the perspective of NHI beneficiaries showed that patients perceived gatekeepers as barriers to accessing advanced healthcare services [7]. Most studies of GPs have focused on their technical competence as primary care physicians and did not specifically assess their gatekeeper role [8–10]. Studies in other countries have found that the gatekeeper role was not always optimally carried out in primary care settings and that this was likely attributable to GP behaviour [11–13]. GPs’ perceptions about gatekeeping may have hampered the effectiveness of primary care facilities in their gatekeeper role, but empirical evidence to support such an assumption is still limited [14–16]. Nor have factors been explored that might be associated with GPs’ perceptions.

Using the example of Indonesia, which has one of the largest primary care networks among low- and middle-income countries, we sought to fill this evidence gap by assessing GPs’ perceptions of the gatekeeper role and investigating associated factors. Specific aims were (i) to examine the domains of knowledge, attitude and performance in order to describe how GPs perceived the gatekeeper role of primary care, (ii) to explore associations of personal and facility characteristics with the knowledge, attitudes and performance of GPs with respect to the gatekeeper role, and (iii) to assess the association between GPs’ knowledge and attitude with the performance of GPs toward the gatekeeper role of primary care.

## Methods

### Study design, setting and population

We conducted a cross-sectional, paper-based survey between March to October 2016, in the Banyumas district, located in the southwest of Indonesia’s Central Java province. With approximately 1.5 million inhabitants, Banyumas is one of the province’s largest and most populated districts. NHI coverage in Banyumas was about 61% of the total population in 2016. By the same year, 75 primary care facilities had been contracted by the district NHI agency to be entry points to the healthcare system and to function as gatekeepers for the NHI beneficiaries in Banyumas. Respondents were recruited based on facilities as the primary sampling unit. For each facility, we targeted the GP who serves as “physician in charge” (clinical director) to obtain a sample size of at least 75 GPs. In addition, we tried to further expand the study sample to get more representation of the GPs by inviting through the clinical director also the other GPs who work in the same facility to participate in the survey. We finally recruited a total of 96 of 365 GPs from all 75 primary care facilities in the district for our study. Two research assistants visited those facilities and distributed and collected the paper-based questionnaires, which were filled in manually by the respondents within a two-week period. From the recruited sample, 81 GPs had agreed to participate and signed an informed consent form (84% response rate). We excluded 8 GPs due to incomplete data, yielding a final sample size of 73 GPs who act as clinical director from 73 primary care facilities which were 97% of the facilities contracted by the NHI agency in Banyumas.

### Measurement

All study data were collected at individual (GPs) level using a tailor-made Indonesian-language questionnaire specially designed for our study. We validated the questionnaire in multiple steps. First, face validity was assessed by two experts in related fields. One was a GP with a certification in family medicine and 15 years’ experience practising at a primary care facility. The other was a GP with professional experience at the Indonesian NHI agency, providing a good understanding of the relationship between primary care facilities and the NHI agency. We used official guideline about gatekeeper function for primary care facilities issued by NHI agency as primary reference to develop the questionnaire. We ended up with twenty provisional items for each of the three questionnaire domains. Second, to assess the construct validity of the questionnaire, we conducted a pilot test with twenty respondents considering the resources constraint, problem prevalence, and power of the test. Using the formula of sample size calculation for the pre-test questionnaire, our relatively small sample size had a study power of more than 80% which was sufficient for a robust pre-test questionnaire [17]. We used the results to implement factor analysis using principal component analysis (PCA) with varimax rotation and Kaiser normalisation. We then excluded items with factor loadings of less than 0.3. Third, following the results from the PCA, we assessed the reliability of the questionnaire by calculating Cronbach’s alpha. We altered the scale-item-deleted combination to maximise the value of alpha (≥ .60) for each questionnaire domain. We concluded that seven items were valid for measuring the knowledge domain, ten items for the attitude domain and seven for the performance domain. The results of the PCA and the reliability analysis, as well as an English translation of the questionnaire, are provided in the supplementary materials to this paper.

We used true–false questions to measure the knowledge domain, with each correct or incorrect answer scored as 1 or 0 respectively. For the attitude domain, we used a two-category rating scale (‘agree‘ and ‘disagree’), with each favourable or unfavourable answer scored as 1 or 0. The performance domain was assessed with questions with a yes/no answering option, indicating whether or not a particular gatekeeping function of primary care was fulfilled as GPs perform their daily duties in serving patients. A similar 1–0 scoring system was applied. Respondents’ final scores per domain were calculated as a percentage of the maximum score for that domain.

On the same questionnaire we collected information about respondents’ personal characteristics and those of the facilities where they worked, to be used as independent variables. We included as personal demographic variables age, gender, type of medical school curriculum (conventional or competency-based), work experience as a GP (less than 5 years, 5–10 years, more than 10 years), nature of employment (full-time, part-time) and employment status (civil service or private).

For facility characteristics, we collected information on location, type of primary care facility and duration of its NHI contract relationship. We categorised location into urban and rural, based on the urban–rural status of the facility’s sub-district as obtained from the district government. Type of primary care facility was categorised into three groups: public primary healthcare centre, private primary care clinic and private physician practice. The first is owned by the district government and usually available in every sub-district. The second are private group or joint practices of GPs and the third are solo GP practices.

### Statistical analysis

To describe the basic characteristics of the survey respondents, we displayed the frequency and percentage of each categorical variable. We tested the distribution of the data using Kolmogorov-Smirnov test and the results indicated that the data were not normally distributed (*p*=0.000)*.* However, we still used means and standard deviations despite the data distribution was not normal to describe the scores in the knowledge, attitude and performance domains, as the differences between mean and median were very small. In bivariate analysis, we compared the scores for knowledge, attitude and performance across various groupings of respondents based on personal and facility characteristics. Considering the data distribution was not normal, we applied two non-parametric tests to compare scores between groups: the Mann–Whitney test for two independent groups and the Kruskal–Wallis test for three independent groups. This analysis was intended to provide an initial description for further exploration of factors associated with scores in each domain.

To assess associations of personal and facility characteristics with the scores on knowledge, attitude and performance, we conducted multivariate analysis using a generalised linear model (GLM). A GLM has more flexible assumptions related to the data distribution than ordinary linear regression, which requires a normal data distribution [18]. We used gamma distribution and identity function in our analysis considering that the value of dependent variable was always positive likely skewed toward larger positive values. We developed two models for the regression analysis so as to provide more consistency in our analysis. In the first model, we included a single independent variable adjusted for age, because age (but not gender) had been identified in bivariate analysis as strongly associated with the scores on each domain. The second model was a full-adjusted model, including all independent variables and adjusted for age. A similar approach was used to assess the association between GPs’ knowledge and attitude and GP’s performance. We provide two basic models which assess the association between a single variable GPs’ knowledge or attitude and a combined variable of GPs’ knowledge and attitude, with GP’s performance. For both models, we adjusted for age only and for all variables including age, personal characteristics, and facility characteristics which made a total of four models in the analysis. The regression coefficient of each independent variable was deemed statistically significant if the *p*-value was less than .05.

## Results

### Characteristics of respondents

Table 1 shows the basic characteristics of the study respondents. Most were male (56.2%) and in the age category between 30 and 50 years (68.5%). Most respondents had been trained under the conventional medical school curriculum (68.5%) and the majority had been practising as GPs for more than 10 years (56.2%). In terms of employment, the majority were working full time (61.6%) and had civil servant status (67.1%). The majority were practising in facilities located in rural areas (69.9%), most worked in public primary healthcare centres (49.3%), and most in facilities contracted by the NHI less than 2 years previously (53.4%).

**Table 1:** Basic characteristics of the survey respondents

	***n***	%
**Personal characteristics**		
**Gender**		
Male	41	56.2
Female	32	43.8
**Age**		
< 30 years	12	16.4
30–50 years	50	68.5
> 50 years	11	15.1
**Medical school curriculum**		
Conventional	50	68.5
Competency-based	23	31.5
**Working experience**		
< 5 years	16	21.9
5–10 years	16	21.9
> 10 years	41	56.2
**Nature of employment**		
Full-time	45	61.6
Part-time	28	38.4
**Civil servant**		
Yes	49	67.1
No	24	32.9
**Facility characteristics**		
**Location**		
Rural	51	69.9
Urban	22	31.1
**Type of primary care facility**		
Public health centre	36	49.3
Private clinic	13	17.8
Private physician practice	24	32.9
**Duration contracted by NHI**		
≤ 2 years	39	53.4
> 2 years	34	46.6

### Knowledge, attitude and performance with respect to the gatekeeper role

We display the respondents’ overall scores on knowledge, attitude and performance, as well as the distribution of scores among various groupings, in Table 2. The average GP knowledge score was 75.1% (SD 20.5). Comparing the knowledge scores among groups, we observed notable differences between age groups and between public and private primary care settings. GPs in the oldest age group scored highest on knowledge, with a mean of 78.4% (SD 18.1), while those practising in private settings had the highest means of around 81%.

**Table 2:** Distributions of GPs’ scores on knowledge, attitude and performance regarding the gatekeeper role of primary care

	Knowledge			Attitude			Performance		
	Mean	SD	***p***	Mean	SD	***p***	Mean	SD	***p***
**Overall**	75.1	20.5		64.5	24.5		78.5	11.2	
**Personal characteristics**									
**Gender**									
Male	75.9	20.3	.65	65.0	21.2	.67	77.3	11.9	.34
Female	74.1	20.9		63.9	21.1		79.9	10.2	
**Age**									
< 30 years	67.9	27.3		52.8	22.2		76.2	7.0	
30–50 years	74.0	19.0	**.04**	64.4	24.9	**.04**	78.9	11.9	.67
> 50 years	88.3	12.5		77.7	19.2		79.2	11.7	
**Medical school curriculum**									
Conventional	76.8	17.9	.52	67.3	25.6	.09	79.7	11.9	.12
Competency-based	71.4	25.1		58.4	21.2		75.7	9.1	
**Working experience**									
< 5 years	67.9	28.3		47.2	22.0		75.0	8.2	
5–10 years	74.1	18.2	.54	65.3	28.4	**.00**	78.6	12.7	.25
> 10 years	78.4	17.2		71.0	20.9		79.8	11.5	
**Nature of employment**									
Full-time	72.7	19.9	.14	62.9	24.6	.44	77.1	11.1	.34
Part-time	79.1	21.1		67.1	24.6		80.6	11.1	
**Civil servant**									
Yes	76.1	18.9	.86	63.5	24.3	.57	79.6	11.3	.18
No	73.2	23.6		66.7	25.3		76.2	10.8	
**Facility characteristics**									
**Location**									
Rural	73.4	20.8	.18	67.1	23.9	.17	77.8	11.5	.40
Urban	79.2	19.6		58.5	25.4		79.9	10.5	
**Type of primary care facility**									
Public health centre	68.6	22.1		58.9	25.1		80.5	10.3	
Private clinic	81.3	14.7	**.03**	67.5	26.6	.16	78.0	15.0	.24
Private physician practice	81.5	18.1		71.3	21.1		75.6	9.8	
**Duration contracted by NHI**									
≤ 2 years	74.3	19.4	.35	64.9	23.9	.89	76.5	10.6	.12
2 years	76.0	21.8		64.1	25.5		80.7	11.6	

In the attitude domain, GPs’ overall average score was 64.5% (SD 24.5). Differences in attitude scores emerged among different age groups and in relation to medical school curriculum, work experience as GPs, and location and type of primary care setting. The highest mean scores were found among GPs in the oldest age group (77.7%, SD 19.2), the conventionally trained (67.3%, SD 25.6), the most experienced (71.0%, SD 20.9), those practising in rural areas (67.1%, SD 24.6) and the solo practitioners (71.3%, SD 21.1).

The GPs’ average overall score for the performance domain was 78.5% (SD 11.2), the highest average score of the three domains. GPs of older age, with more work experience, in urban practice, and practising in public facilities tended to have higher scores on performance than those in the respective comparison categories, but the differences were relatively small.

### Factors associated with GPs’ knowledge, attitudes and performance as gatekeepers

In Table 3, we explore personal and facility characteristics as factors potentially associated with GPs’ scores in terms of knowledge, attitude and performance with regard to the primary care gatekeeping role. For knowledge scores, we found no personal or facility characteristics with significant associations in either Model 1 or 2, although GPs in private practice tended to score higher on knowledge than those working in public primary healthcare centres (*B* = 10.27, *p* = .06; Model 2).

**Table 3:** Factors associated with GPs’ scores on knowledge, attitude and performance regarding the gatekeeper role of primary care

	Knowledge				Attitude				Performance			
	Model 1		Model 2		Model 1		Model 2		Model 1		Model 2	
	***B***	***p***	***B***	***p***	***B***	***p***	***B***	***p***	***B***	***p***	***B***	***p***
**Personal characteristics**												
**Medical school curriculum**												
Conventional	Ref	–	Ref	–	Ref	–	Ref	–	Ref	–	Ref	–
Competency-based	0.29	.99	−0.31	.96	−1.27	.87	−5.50	.43	−4.40	.23	−4.72	.17
**Working experience**												
< 5 years	Ref	–	Ref	–	Ref	–	Ref	–	Ref	–	Ref	–
5–10 years	5.35	.55	7.10	.45	25.00	**.01**	22.10	**.00**	5.10	.31	7.59	.14
> 10 years	5.92	.49	3.49	.70	28.93	**.00**	32.20	**.00**	6.68	.18	6.91	.16
**Nature of employment**												
Full-time	Ref	–	Ref	–	Ref	–	Ref	–	Ref	–	Ref	–
Part-time	6.88	.14	5.23	.31	5.34	.34	2.13	.70	3.99	.13	6.81	**.01**
**Civil servant**												
Yes	Ref	–	Ref	–	Ref	–	Ref	–	Ref	–	Ref	–
No	−5.53	.33	−4.72	.44	5.64	.41	15.13	**.02**	−3.69	.25	0.65	.84
**Facility characteristics**												
**Location**												
Rural	Ref	–	Ref	–	Ref	–	Ref	–	Ref	–	Ref	–
Urban	0.90	.87	3.77	.53	−17.31	**.00**	−14.05	**.03**	2.54	.62	4.12	.20
**Type of primary care facility**												
Public health centre	Ref	–	Ref	–	Ref	–	Ref	–	Ref	–	Ref	–
Private clinic	10.65	.10	10.26	.14	6.79	.15	4.87	.52	−3.40	.36	−4.73	.20
Private physician practice	10.24	.05	10.27	.06	9.40	.39	8.17	.17	−6.03	**.04**	−6.00	**.04**
**Duration contracted by NHI**												
≤ 2 years	Ref	–	Ref	–	Ref		Ref	–	Ref	–	Ref	–
> 2 years	−0.81	.86	1.32	.70	−3.57	.52	1.14	.82	4.17	.11	3.69	.15

Model 1: each factor, adjusted for age; Model 2: all factors included, adjusted for age

In the attitude domain, GPs’ work experience and facility location were consistently associated with their scores in both models. In Model 2, our finding showed that GPs practising 5 to 10 years (*B* = 22.10, *p* = .00) and more than 10 years (*B* = 32.20, *p* = .00) scored higher than those practising less than 5 years. Privately employed GPs were likely to have higher attitude scores than those with civil servant status (*B* = 15.13, *p* = .02). GPs practising in urban areas were likely to score lower in attitude than those in rural areas (*B* = − 14.05, *p* = .03).

In terms of performance, our findings showed that part-time employment was associated with a higher score than full-time work (*B* = 6.81, *p* = .01; Model 2). With regard to facility characteristics, employment in private practice was associated with lower performance scores than employment a public primary healthcare centre (*B* = − 6.00, *p* = .04).

### The association between GPs’ knowledge and attitude with GPs’ performance as gatekeepers

In Table 4, we displayed the association between GPs’ knowledge and attitude with GPs’ performance as gatekeepers. In all four models, GPs’ knowledge consistently was not associated with GPs’ performance. For attitude, our findings showed that GPs’ attitude was positively associated with the GPs’ performance as gatekeeper. In three of four models, the regression coefficients were statistically significant. After adjusted for all variables, a higher score of GPs’ attitude was associated with higher score of GPs’ performance as gatekeepers (B = 0.160, *p* = .00) as shown in the fourth model.

**Table 4:** Association between knowledge and attitude of GPs’ with the performance of GPs as gatekeepers

	Model 1		Model 2		Model 3		Model 4	
	***B***	***p***	***B***	***p***	***B***	***p***	***B***	***p***
**Knowledge**	−0.065	0.32	−0.075	0.23	−0.096	0.11	−0.114	0.06
**Attitude**	0.101	0.64	0.137	0.01	0.118	0.03	0.160	0.00

Model 1: Knowledge or attitude adjusted to age; Model 2: Knowledge or attitude adjusted to age, personal characteristics, and facility characteristics; Model 3: Knowledge and attitude adjusted to age; Model 4: Knowledge and attitude adjusted to age, personal characteristics, and facility characteristics

## Discussion

Our study described how general practitioners (GPs) in the Indonesian Banyumas district perceive the gatekeeper role of primary care and it explored whether those perceptions were associated with personal characteristics of the GPs or with characteristics of the facilities where they worked. Findings showed that GPs scored relatively high in the domains of knowledge and performance, but lower in the attitude domain. No personal or facility characteristics were associated with GPs’ knowledge about the primary care gatekeeper function. Longer work experience and private rather than civil service employment were associated with higher attitude scores, and urban practice location with lower scores. Part-time employment was associated with higher scores on gatekeeping performance, while private practice was linked to lower scores. Higher scores of GPs’ attitude were associated with GPs’ performance as gatekeepers, while GPs’ knowledge showed no association with GPs’ performance as gatekeepers.

### Strengths and limitations

To the best of our knowledge, this is the first study in Indonesia to explore how GPs perceive the gatekeeper role of primary care and the factors associated with their perceptions. Only a few studies have assessed the physician perspective on primary care in Indonesia, with most prior studies focusing on the patients’ point of view. Our study sought to provide new insights and initial evidence on key areas that can facilitate primary care in performing its gatekeeping role.

Our study is not without limitations. Its relatively small sample size may raise concern about the power of the study and the robustness of our estimation. However, given the exploratory nature of our study and the flexibility of the statistical technique we employed in the data analysis, we believe the internal validity can be considered adequate. The sample size may also imply limited generalisability for our findings. We consider our study site (Banyumas district) to bear a good resemblance – in terms of demographics and socioeconomic, cultural and geographical background – to the majority of districts on the islands of Java and Sumatra, where around 70% of the Indonesian population lives. But the findings should be interpreted cautiously with regard to generalisability. Another issue is related to sample size for the validity test which may less than usually used in other studies. We acknowledge that resources constraint hindered our ability to reach a larger sample size for the questionnaire pre-test. However, our relatively small pre-test sample size has been calculated properly based on a robust method. Therefore, our sample size had sufficient power required for a valid questionnaire pre-test.

### Interpretation

The findings indicated that GPs generally understood and carried out the gatekeeper role adequately, although they perceived the role less positively. Their less positive attitude was likely related to a perception that gatekeeping formed an additional burden in their daily duties. A study in Indonesia has found that GPs perceive increasing demand from patients as one of the barriers to performing optimally as primary care physicians [10]. Moreover, it has been reported that patients perceive the quality of primary care as low and that they view the gatekeeper as an access barrier to more advanced services perceived to be of higher quality [7]. This combination of gatekeeping-related factors likely leads to more tension in the doctor–patient relationship and a more stressful work environment for GPs. The gatekeeper role may also be perceived by GPs to be in conflict with their professional autonomy [19]. As part of that role, GPs are expected to consider additional aspects in their clinical decision making, such as costs for the patient. Studies in other countries have shown that physicians have negative attitudes to policies they perceive as interventions in their professional autonomy, particularly when those are based on economic motives [20–22].

We found no personal or facility characteristics that were significantly associated with the GPs’ knowledge about the gatekeeper function of primary care, although privately employed GPs tended to higher knowledge scores than those practising in public facilities. This was likely associated with the financial incentives system currently employed by the NHI agency. Primary care providers are paid by the agency under a capitation system, with payments adjusted to their performance as gatekeepers [4]. For private clinics and practices that rely mainly on those payments as their revenue source, that system will motivate them to a better understanding of the gatekeeper role. In public facilities, where most practising GPs are civil servants with salaries paid from government budgets, the motivation to understand the gatekeeper role may be lower, because there are no direct consequences for financial rewards. Although no studies have documented such an association in Indonesia, studies elsewhere have reported strong associations between financial incentives and physicians’ perceptions about the implementation of policies in practice settings [22, 23].

We observed that GPs who had longer work experience and those who were privately employed had more positive attitudes towards the primary care gatekeeper function. The more experienced GPs may have been exposed to more learning activities, such as specific training that shapes their attitudes more positively towards specific roles or issues such as gatekeeping [24, 25]. Just as with the gatekeeping knowledge scores of private providers, financial incentives may play a part in the higher attitude scores of GPs in private employment. The rapid increases in NHI coverage have prompted more private providers to rely on NHI capitation funding as their main revenue sources. However, competition among private providers is relatively high, as they are allowed to register only non-subsidised NHI beneficiaries. Subsidised (lower-income) beneficiaries are automatically registered at the nearest public primary healthcare centre [4]. Given that NHI capitation is calculated on a per capita basis and adjusted to the performance of private providers, including in gatekeeping, this arguably influences privately employed GPs to have more positive attitudes towards the gatekeeper function. It also implies a potential of private providers to provide better primary care services in terms of quality, access and efficiency, presuming the right incentives are forthcoming from government.

The association between urban GPs and low gatekeeper attitude scores may be explained by differences in the composition of the populations of NHI beneficiaries who live in rural and urban areas. The majority in urban areas are non-subsidised beneficiaries of relatively high socioeconomic status [3]. This segment consists mostly of workers in formal economic sectors who are known to be more demanding, having enjoyed more flexible benefits from their previous insurance schemes before these were subsumed under the NHI [26]. Perceiving primary care to be of low quality, urban patients often request direct referral to secondary care facilities [7]. Currently, the strict regulations applying to NHI beneficiaries for accessing healthcare curtail such swift referrals and lead to more tension in physician–patient relationships [10, 27]. Such frequent conflicts may generate additional stress for GPs, thereby inducing more negative attitudes towards the gatekeeping function. These critical issues need to be urgently addressed, in order to avoid a further negative impact on the geographical accessibility of primary care (urban–rural disparities), on perceived quality due to low patient satisfaction, and on low efficiency caused by unnecessary referrals to secondary care.

The association between part-time employment status and higher scores on gatekeeping performance can be explained by the characteristics of part-time working relationships in Indonesian GP practice. It is legal, and common practice, that GPs work at multiple sites (up to three different facilities) [3]. Such GPs usually practise in one main facility in full-time employment and part-time in one or more other facilities. In facilities where GPs are working part-time, the working arrangements between the facilities and GPs are usually more flexible [10]. Such an environment reduces the work pressure of the GPs [9], which arguably may favourably influence their performance in carrying out the gatekeeping function.

Our finding also showed that GPs in private practice were likely to score lower on gatekeeping performance. This may be attributable to the different practice standards applied by the NHI agency to this type of practice. The NHI agency has contracted private practices because of the inadequacy of other primary care facilities to cope with the additional demand for healthcare that arose after the rapid expansion of NHI coverage [28]. However, the required standards in terms of services such as supporting equipment or office hours are somewhat lower than those for the public and private clinics [4]. This may impair the private GPs’ ability to carry out of the gatekeeper function. Moreover, private practice physicians usually work in solo GP practices without specific support from a management team. This may considerably impede implementation of the gatekeeper function in comparison with healthcare facilities that have monitoring and evaluation mechanisms in place to ensure that the function is adequately carried out. A systematic review of studies in low- and middle-income countries has found that individual private healthcare providers showed lower adherence to regulations than institutional providers [29].

In our final analysis, our study revealed that GPs’ attitude but not their knowledge had a positive association with GPs’ performance as gatekeepers. In many previous studies that assessed the association between GPs’ knowledge with their practice in several issues such as clinical guideline implementation, those associations were rarely found [30, 31]. Knowledge depends more on personal characteristics and can be straightforwardly improved, while performance is largely influenced by contextual factors which often go beyond individual capability and may become significant barriers for translation of knowledge into practice [32]. For attitude, its linear associations with performance was commonly found in many studies that investigate GPs’ behaviour related to the care provision in primary care settings [33, 34]. Attitude reflects individual’s favourable or unfavourable feeling towards a particular issue and forms a strong driving factor for behaviours as much as others facilitating factors (e.g. contextual factors) permit [35].

## Conclusions and policy implications

Our study demonstrates that GPs in Indonesia are knowledgeable and that they adequately carry out the function of gatekeeper in primary care. However, their attitude towards that function is less positive. Contextual factors such as location and type of facility likely play a major role in influencing GPs’ perceptions towards the gatekeeper function, although individual characteristics such as work experience might also be determining factors. Considering a longer working experience may determine the attitude of GPs toward gatekeeper role, a continued support to GPs which create a more positive working experience may improve the attitude of GPs toward the gatekeeper role. These include routine continuing medical education (CME) and continuing professional development (CPD). Since GPs in urban areas had lower attitude scores and this likely related to a more tense doctor-patient relationship in urban setting, effort to improve the doctor-patient relationship in the context or gatekeeping function become crucial. This includes a social marketing programme about NHI gatekeeping regulations which targeted particularly amongst higher-income NHI beneficiaries living in urban areas. As private providers particularly GP solo practice scored lower in performance toward gatekeeper role, policies to support the improvement of practice standards in private practices particularly for solo GP practice may be useful in increasing their compliance with the gatekeeper function of primary care in the current system. Future studies which use more comprehensive approaches such as a mix-method may provide a better insight on the association between knowledge, attitude, and performance of GPs toward gatekeeper role in Indonesia.

## Supplementary Information

**Additional file 1: Table S1.** Results of principal component analysis. **Table S2.** Results for reliability analysis**.**

## Data Availability

The dataset used and analysed in this study is available from the corresponding author on reasonable request.

## Abbreviations

GP: General practitioner
NHI: National health insurance
GLM: Generalised linear model
CME: Continuing medical education
CPD: Continuing professional development
