# Supplementary material for: Exploring general practitioners’ perceptions about the primary care gatekeeper role in Indonesia
Source: BMC Fam Pract. 2021 Jan 4;22:5. doi: 10.1186/s12875-020-01365-w (PMC7780672; doi:10.1186/s12875-020-01365-w)
Supplement: Supplementary file 1 — Additional file 1: Table S1. Results of principal component analysis. Table S2. Results for reliability analysis. [file 12875_2020_1365_MOESM1_ESM.pdf]

Table S1. Results of principal component analysis

Item number	Factor loading		
	Knowledge	Attitude	Practice
1	0.668	-0.236	-0.236
2	0.705	0.082	0.082
3	n.a	0.651	0.651
4	0.139	-0.280	-0.280
5	-0.267	-0.142	-0.142
6	0.048	0.159	0.159
7	0.529	-0.585	-0.585
8	-0.207	-0.448	-0.448
9	-0.018	n.a	0.492
10	-0.233	0.492	-0.080
11	0.538	-0.080	0.234
12	0.519	0.234	-0.104
13	0.232	-0.104	0.426
14	0.323	0.426	0.296
15	0.117	0.296	0.727
16	-0.165	0.727	0.757
17	0.408	0.757	0.656
18	-0.031	0.656	0.640
19	0.230	0.640	0.318
20	-0.403	0.318	-0.236

Note: Only item with factor loading ≥ 0.3 were included in the following analysis, n.a: not available due to no variation

Table S2. Results for reliability analysis.

Domain	Item included in analysis	Cronbach's alpha
Knowledge	1, 2, 7, 11, 12, 14, 17	0.609
Attitude	3, 8, 10, 14, 16, 17, 18, 19, 20	0.654
Practice	3, 7, 8, 13, 16, 18, 19	0.608

Note: some items from principal component analysis were deleted to get the largest value of Crobach's alpha

Research Questionnaire [translated from Indonesian version]

No.	Item	Response
	**A. Respondent characteristics**	
1.	What is your gender?	a. Male
		b.Female
2.	What is your age	Year
3.	How long have you been practising as a GP?	Year
4.	In what year did you graduate from medical school?	
5.	In which university you were trained as a physician?	
6.	What kind of medical curriculum have you experienced in your training?	a. Conventional
		b. Competency-based
7.	Are you currently working full-time as a GP in this facility?	a. Yes
		b. No
8.	Are you also practising as a GP in other facilities?	a. Yes
		b. No
9.	If yes, in which facilities? (can be more than 1 response)	a. Public healthcare centre
		b. Private clinic
		c. Private solo practice
		d. Public hospital
		e. Private hospital
		f. Others
10.	Are you a civil servant in public healthcare facilities?	a. Yes
		b. No
11.	Are you a civil servant working in other public institutions? (e.g. public university)	a. Yes
		b. No
12.	Are you a full-time employee in private healthcare facilities?	a. Yes
		b. No
13.	What is your current position in this facility?	a. Person in-charge
		b. Person in-charge and owner
	**B. Facility characteristics**	
1.	Location	
	a. Street	
	b. Village	
	c Subdistrict	
2.	Type of facility	a. Public healthcare centre
		b. Military/police clinic
		c. Private solo practice
		d. Private clinics
		e. Type D hospital
		f. Others
3.	How long has this facility been a provider for the National Health Insurance?	months

4.	How long have you been the person-in-charge of this facility?	months
5.	Number of NHI beneficiaries registered at this facility	persons
6.	Number of full-time GPs in this facility	persons
7.	Number of full-time nurses in this facility	Persons
8.	Number of full-time non-health personnel in this facility	Orang
9.	Is this facility providing 24 hours service?	a. Yes
		b. No
10.	If not, how many hours a day is does this facility provide services (opening hours)?	Hours
11.	Is this facility providing inpatient care service?	a. Yes
		b. No
	**C. Knowledge about gatekeeping function.**	
1.	Have you ever heard of the term 'gatekeeper'?	a. Yes
		b. No
2.	Do you understand what the function of gatekeeper entails?	a. Yes
		b. No
3.	What kind of healthcare facilities have a role as gatekeeper?	a. Public health centre
		b. Type C hospital
		c. Type B hospital
		d. Type
4.	Every NHI beneficiary must be registered only in a single primary care facility.	a. True
		b. False
5.	In emergency cases, NHI beneficiaries can directly seek care at a type C hospital.	a. True
		b. False
6.	Primary care facilities can provide a referral to secondary care facilities based on a patient's request.	a. True
		b. False
7.	Primary care facilities must provide cervical cancer screening as part of their services	a. True
		b. False
8.	Primary care facilities must provide basic child immunisation as part of their services	a. True
		b. False
9.	Primary care must organise comprehensive management for patients with hypertension.	a. True
		b. False
10.	If a patient visits a primary care facility with great chest pain, what should the GP do?	a. Catheterization
		b. Prescribe thrombolytic
		c. Coronary by pass
		d. Prescribe nitrate
11.	GPs must perform home visits when patient is unable to travel to primary care facilities.	a. True
		b. False

12.	Primary care facilities provide long-distance consultations (hotline).	a. True
		b. False
13.	Medical records from patients who are in the same family can be kept in one folder.	a. True
		b. False
14.	How long is the average consultation time for a patient who visits a primary care facility?	a. 5 minutes
		b. 10 minutes
		c. 15 minutes
15.	What is the ideal GPs ratio to the number of NHI beneficiaries in primary care facilities?	a. 1:2000
		b. 1:3000
		c. 1:5000
16.	Primary care facilities can directly provide medicine to the patient without involving pharmacies(self-dispensing)	a. True
		b. False
17.	What is the main measure to indicate whether the function of gatekeeper is performed in primary care?	a. Patient satisfaction
		b. Waiting time
		c. Mortality rate
		d. Number of referrals
18.	GPs should routinely monitor patients with type 2 diabetes	a. True
		b. False
19.	Patients with post ischaemic stroke in a stable condition should have a routine check at neurology clinics in hospital.	a. True
		b. False
20.	GPs should do home visits for patients who have been recently discharged from the hospital	a. True
		b. False
	**C. Attitude toward gatekeeping**	
1.	Primary care facilities have the ability and resources to carry out the gatekeeper function	a. Agree
		b. Disagree
2.	Primary care can solve most of patient's health needs	a. Agree
		b. Disagree
3.	The gatekeeper function adds a burden of GPs in primary care facilities.	a. Agree
		b. Disagree
4.	Patients who only ask for a referral when they a visit primary care facility, do not acknowledge the competence of GPs	a. Agree
		b. Disagree
5.	GPs should actively support patients with achieving a healthy lifestyle.	a. Agree
		b. Disagree
6.	Primary care facilities who perform the gatekeeper function well will contribute to reducing healthcare costs	a. Agree
		b. Disagree

7.	Patients often do not fully trust GPs' competence to solve their health problem.	a. Agree
		b. Disagree
8.	Patients often express dissatisfaction after they receive treatment from GPs	a. Agree
		b. Disagree
9.	GPs should continually update their competence	a. Agree
		b. Disagree
10.	The workload in primary care facilities lead to little opportunities for GPs to update their competence	a. Agree
		b. Disagree
11.	Primary care facilities and hospitals are equal partners.	a. Agree
		b. Disagree
12.	Hospitals often did not provide a return referral to primary care facilities for patients who just finished their treatment in the hospital.	a. Agree
		b. Disagree
13.	Patients can easily access primary care	a. Agree
		b. Disagree
14.	Primary care facilities compete with each other	a. Agree
		b. Disagree
15.	Primary care facilities frequently communicate with each other and share information	a. Agree
		b. Disagree
16.	NHI agency provides adequate support for primary care facilities to carry out the gatekeeper function	a. Agree
		b. Disagree
17.	NHI agency treats primary care facilities as an equal partner to provide healthcare for beneficiaries	a. Agree
		b. Disagree
18.	NHI agency acknowledges the efforts made by primary care facilities to carry out the gatekeeper function	a. Agree
		b. Disagree
19.	Communication between the NHI agency and primary care facilities is well maintained	a. Agree
		b. Disagree
20.	NHI agency often takes the patient's side when there is a conflict between primary care facilities and patients	a. Agree
		b. Disagree
	**D. Practice of gatekeeper function**	
1.	Gatekeeper function has been well carried out in primary care facilities where I work	a. Yes
		b. No
2.	When I see patients, I provide curative care, as well as promotive and preventive care.	a. Yes
		b. No
3.	All GPs working in this facility have met all legal requirements (competence certificate, registration, and license)	a. Yes
		b. No
4.	I have a certification as a family physician	a. Yes

		b. No
5.	I received training in Advanced Trauma Life Support	a. Yes
		b. No
6.	I received training in Advanced Cardiovascular Life Support	a. Yes
		b. No
7.	I have received other advanced medical training (medical emergency, occupational health)	a. Yes
		b. No
8.	I work more than 8 hours per day in this facility	a. Yes
		b. No
9.	I never provide a referral based on patient's request.	a. Yes
		b. No
10.	Primary care facility where I am working provides 24-hour services	a. Yes
		b. No
11.	Primary care facility where I am working have a hotline	a. Yes
		b. No
12.	I do home visits for patients who cannot travel to the facility	a. Yes
		b. No
13.	This facility organises patient medical records based on family order	a. Yes
		b. No
14.	I provide on average 15 minutes consultation time for each patient.	a. Yes
		b. No
15.	This facility is equipped by a medical laboratory	a. Yes
		b. No
16.	This facility has a pharmacy	a. Yes
		b. No
17.	There is a NCDs management programme in this facility	a. Yes
		b. No
18.	The facility where I work has a good collaboration with secondary care facilities	a. Yes
		b. No
19.	The facility where I work is cooperating well with other primary care facilities	a. Yes
		b. No
20.	This facility actively participates in the capacity building programme provided by NHI agency	a. Yes
		b. No

Note: The questionnaire items were developed based on the official guideline for gatekeeper function of primary care facility issued by Indonesian NHI Agency.
